# Pulmonary Aspiration During Procedural Sedation for Colonoscopy Managed With Two Endotracheal Tubes and A McGrath Laryngoscope

**DOI:** 10.7759/cureus.26601

**Published:** 2022-07-06

**Authors:** Sengottaian Sivakumar, Mark J Young, Bhavya Krishna, Roni Mendonca

**Affiliations:** 1 Anesthesiology, Metropolitan Hospitals, New York City, USA; 2 Anesthesiology, Vardhman Mahavir Medical College and Safdarjung Hospital, Delhi, IND; 3 Anesthesiology, Metropolitan Hospital Center, New York City, USA

**Keywords:** perioperative pulmonary aspiration, pulmonary aspiration management, aspiration in ambulatory surgery, aspiration during endoscopy, aspiration management, mcgrath laryngoscope

## Abstract

Perioperative pulmonary aspiration of regurgitated gastric contents is the presence of gastric contents in the tracheobronchial tree. It is diagnosed by direct examination of the airway, bronchoscopy of the tracheobronchial tree, or postoperative imaging which reveals previously not identified lung infiltrates. Our case report describes a novel and successful method to manage perioperative pulmonary aspiration.

## Introduction

Perioperative pulmonary aspiration of regurgitated gastric contents is defined as the presence of gastric or bilious secretions in the tracheobronchial tree [1]. Diagnosis is usually made by direct examination of the airway, bronchoscopic assessment of the tracheobronchial tree, or postoperative imaging that demonstrates lung infiltrates not previously identified on a preoperative radiograph. In a recent closed claims analysis consisting of 115 cases of pulmonary aspiration, death occurred in 57% of the claims and severe permanent injury in another 14% [2]. Here, we report a case of perioperative pulmonary aspiration managed successfully with two endotracheal tubes (ETTs) and a McGrath video laryngoscope (MVL).

## Case presentation

A 69-year-old male with a past medical history of essential hypertension and class I obesity presented to the emergency department after two days of diffuse abdominal pain and obstipation. He also reported one episode of non-bloody, non-bilious vomiting on the day of arrival. Abdominal X-ray revealed dilated large bowel. Abdominal computed tomography showed a possible circumferential mass lesion or stricture at the distal sigmoid colon and a distended proximal colon; the small intestine and stomach were collapsed. Two months prior, the patient had similar complaints and was managed with endoscopic decompression under intravenous sedation. The patient’s vital signs were within normal limits. His physical examination showed a soft, protuberant abdomen which he felt was his baseline and attributed to obesity. The operating surgeon planned to do another endoscopic decompression and requested monitored anesthesia care. The patient had an in-situ nasogastric tube, and approximately 60 ml of clear gastric fluid was suctioned before starting sedation for the planned procedure.

**Figure 1:**
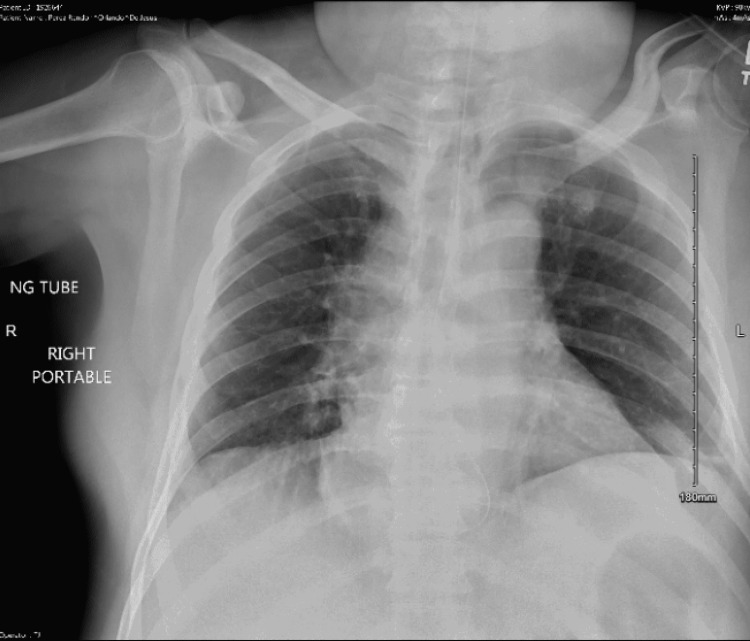
Chest X-ray on postoperative day one

**Figure 2:**
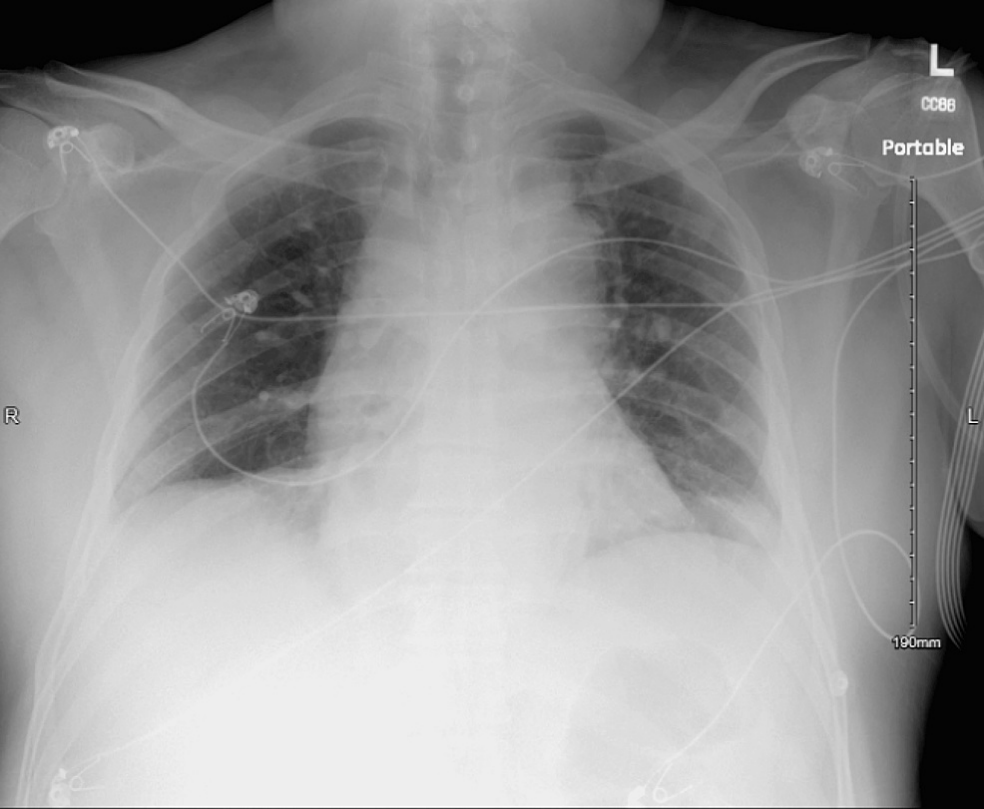
Chest X-ray on postoperative day four showing bilateral pulmonary infiltrates

With the patient in the left lateral position, intravenous sedation was initiated with a bolus of 1 mg/kg propofol. Moments later, profuse bilious secretions started to emanate from the patient’s mouth and nares. He developed tachycardia with a heart rate of 130-140 per minute, oxygen saturation dropped to 84%, and tachypnea developed at a rate of 22-24 breaths per minute. The stretcher was promptly tipped into a steep Trendelenburg position while maintaining the patient’s left lateral positioning. The anesthesia team then attempted endotracheal intubation with an MVL, but the laryngeal view was obfuscated by aspirate and the vocal cords could not be visualized. A 7.5 mm ETT was inserted deliberately into the esophagus, and the cuff was inflated. This prevented more gastric contents from coming out of the esophagus. After suctioning the laryngopharynx above the esophageal ETT cuff, MVL revealed a clear view of the vocal cords, and a second ETT was inserted into the trachea easily. Thorough tracheal suctioning was performed through the tracheal ETT using a 10 Fr catheter with a controlled suction port. Oxygen saturation improved to 94% with positive pressure ventilation and stabilized. Fiberoptic bronchoscopy revealed light soiling of the infraglottic trachea with scant bilious secretions that did not visually extend beyond the main carina. As the patient’s hemodynamics further stabilized, the surgical team proceeded with endoscopic decompression. The patient was extubated at the end of the procedure. Post-extubation saturation remained at 94% with supplemental oxygen through a non-rebreathing mask. The patient was monitored and treated in the surgical intensive care unit, including empiric antibiotics (piperacillin and tazobactam for five days). Immediate post-procedure chest X-ray showed subsegmental atelectasis (Figure 1), and a repeat study on a postoperative day (POD) 4 showed bibasilar pulmonary infiltrates (Figure 2).

The patient felt to have recovered from aspiration by day eight and he was able to complete a 6-minute walk test without any desaturations.

## Discussion

Pulmonary aspiration can cause significant morbidity and mortality. As part of preoperative planning, it is the responsibility of the anesthesiologist to assess a patient’s risk for aspiration and determine an ideal anesthetic plan that considers the risk of pulmonary aspiration. When that risk is elevated, mitigation strategies like strict adherence to NPO (nothing by mouth) guidelines and performing pre-induction gastric suctioning via oral or nasogastric tube can be employed. In certain situations, such as intestinal obstruction, the risk of aspiration remains a concern despite such risk mitigation strategies The case described herein is one such instance.

There is controversy about whether to remove an existing nasogastric tube before induction of sedation or anesthesia. One perspective advocates using the nasogastric tube (NGT) as a “venting mechanism” for reducing intragastric pressure during induction [3]. On the other hand, others feel the gastric tube may stent open the lower esophageal sphincter (LES), which may promote aspiration. In this case, the NGT was left in place and pulmonary aspiration occurred despite appropriate fasting and pre-induction NGT suctioning. One study indicates that the volume of fluid obtained by gastric suctioning correlates poorly with residual gastric volume [4]. It is possible that the in-situ NGT was obstructed by solid gastric contents or abdominal mucosa. Decompression of the stomach before induction might have offered a false sense of security in this context. In our patient, the presence of possibly obstructed NGT may have decreased the barrier pressure and assisted in the regurgitation of gastric contents [5].

During preoperative planning, general anesthesia with rapid sequence induction was considered. However, the patient had been managed for the same issue recently using sedation uneventfully. Also, the stomach was felt to have been decompressed with the in-situ nasogastric tube prior to starting sedation. The canister revealed clear gastric fluids which suggested a lack of particulate matter. Authors speculate that had rapid sequence induction been used, the patient may have still aspirated. The outcome may have been worse in this scenario because of supine (as opposed to left lateral decubitus) positioning. Awake fiberoptic intubation for the purpose of avoiding aspiration was also considered, but that risk was felt to be adequately minimized for the aforementioned reasons and sedation was ultimately chosen.

Management of perioperative aspiration includes all measures to limit exposure of tracheobronchial epithelium to acidic gastric contents and restore pulmonary function to normal as soon as possible. In our patient, immediate Trendelenburg positioning and tracheal suctioning limited the quantity of aspirate and helped the patient recover fast from pulmonary injury. Bile acids and gastric contents cause inflammation of bronchoalveolar surfaces, derange the lipid surface barrier, and compromise the bronchoalveolar innate immunity. Damage to the mucosa of the tracheobronchial tree occurs within seconds, but bronchial secretions neutralize the aspirated acid within minutes [5]. In this case, aspirated gastric contents caused asphyxia by physical obstruction evidenced by a decline in oxygen saturation to 84%. Once the trachea was intubated and suctioned well, oxygen saturation improved to 94% but did not reach the baseline. This indicates a significant alveolar epithelial injury-caused ventilation-perfusion mismatch. Prophylactic use of antibiotics in cases of aspiration is not generally recommended because it may cause an overgrowth of drug-resistant organisms in patients with uncomplicated chemical pneumonitis [6]. However, empirical antibiotic therapy is appropriate for patients who suffered aspiration with preexisting bowel obstruction [6]. Our patient’s condition improved after five days of broad-spectrum empiric antibiotics. Even though bronchoscopic examination after an episode of aspiration is not routinely indicated, we attempted flexible bronchoscopic examination to clear obstruction secondary to aspirated contents. Nevertheless, no attempts were made to neutralize the acid aspirate with saline or bicarbonate lavage, which were proven to increase the damage [6]. Hypoxia also caused tachypnea and tachycardia, which persisted in our patient for two days. However, his oxygen saturation returned to baseline after two days after the incident. His hypoxia even triggered postoperative atrial fibrillation.

The fasting state may be unreliable in elderly people with poor awareness, in children, and in cases of delayed stomach emptying, as in patients with intestinal obstruction or multiple traumas [7]. A new prediction model for assessing gastric volume based on point of care sonographic measurements allows assessing the size and type of stomach contents [8]. Identifying high-risk patients by gastric ultrasound and performing preemptive gastric emptying by naso/orogastric tube reduces the risk of aspiration. If the patient is suspected to be difficult intubation with an increased risk of aspiration awake tracheal intubation is recommended [9].

The first attempt to intubate the trachea was difficult due to profuse gastric contents efflux, so we deliberately intubated the esophagus and inflated the cuff to stop and divert the efflux of gastric contents, which prevented extensive damage to the alveolar epithelium. McGrath laryngoscope is a cable-free design with an attached 2.5-inch screen for the enhanced view, was used to intubate this patient in a left lateral Trendelenburg position. Conventional direct laryngoscopy or video laryngoscopy would have made intubation difficult with a laterally lying patient in Trendelenburg’s position. Using McGrath laryngoscope instead of conventional direct laryngoscopy optimized the intubation time required and thereby, the quantity of pulmonary aspiration was minimized. No such procedures have been reported so far. The swift action of the anesthesiologist prevented diffuse pulmonary soiling.

## Conclusions

Regurgitation or aspiration should always be considered immediately in any spontaneously breathing patient who suffers desaturation, laryngospasm, airway obstruction, bronchospasm, bradycardia, or cardiac arrest. Flexible bronchoscopy turns out to be a valuable tool for managing perioperative aspiration. However, anesthesiologists have limited training and experience in clearing luminal obstructions endoscopically. Our case report describes a novel method for managing aspiration successfully. It prompts anesthesiologists to learn to clear luminal obstructions endoscopically.
